# PDGF-C Induces Maturation of Blood Vessels in a Model of Glioblastoma and Attenuates the Response to Anti-VEGF Treatment

**DOI:** 10.1371/journal.pone.0005123

**Published:** 2009-04-08

**Authors:** Emmanuelle di Tomaso, Nyall London, Daniel Fuja, James Logie, James A. Tyrrell, Walid Kamoun, Lance L. Munn, Rakesh K. Jain

**Affiliations:** E.L. Steele Laboratory for Tumor Biology, Department of Radiation Oncology, Massachusetts General Hospital and Harvard Medical School, Boston, Massachusetts, United States of America; Cleveland Clinic, United States of America

## Abstract

**Background:**

Recent clinical trials of VEGF inhibitors have shown promise in the treatment of recurrent glioblastomas (GBM). However, the survival benefit is usually short-lived as tumors escape anti-VEGF therapies. Here we tested the hypothesis that Platelet Derived Growth Factor-C (PDGF-C), an isoform of the PDGF family, affects GBM progression independent of VEGF pathway and hinders anti-VEGF therapy.

**Principal Findings:**

We first showed that PDGF-C is present in human GBMs. Then, we overexpressed or downregulated PDGF-C in a human GBM cell line, U87MG, and grew them in cranial windows in nude mice to assess vessel structure and function using intravital microscopy. PDGF-C overexpressing tumors had smaller vessel diameters and lower vascular permeability compared to the parental or siRNA-transfected tumors. Furthermore, vessels in PDGF-C overexpressing tumors had more extensive coverage with NG2 positive perivascular cells and a thicker collagen IV basement membrane than the controls. Treatment with DC101, an anti-VEGFR-2 antibody, induced decreases in vessel density in the parental tumors, but had no effect on the PDGF-C overexpressing tumors.

**Conclusion:**

These results suggest that PDGF-C plays an important role in glioma vessel maturation and stabilization, and that it can attenuate the response to anti-VEGF therapy, potentially contributing to escape from vascular normalization.

## Introduction

Glioblastoma Multiforme (GBM) is a uniformly fatal tumor afflicting approximately 9,000 persons each year in the United States, and there is currently no efficacious therapy. Standard treatment is maximal resection, combined daily temozolomide and radiation (chemoradiation) followed by 6 monthly cycles of post-radiation temozolomide alone. Unfortunately, survival after recurrence is only a few months [1]. Anti-VEGF treatment of recurrent tumors has shown some promise, but these tumors invariably escape VEGF-blockade [2], [3], [4]. Thus novel targets are desperately needed to guide the development of more effective, innovative therapeutic approaches.

Platelet-derived growth factors (PDGF) are a pleiotropic family of peptides that signal through cell surface, tyrosine kinase receptors (PDGFR) and stimulate cellular functions including growth, proliferation and differentiation [5]. The PDGF family was initially discovered as a mitogen in human serum, localized in the alpha-granules of platelets [6]. The biological role of PDGF signaling in solid tumors can vary from autocrine stimulation of cancer cell growth [7], [8] to subtler paracrine interactions involving adjacent stroma [9], [10], [11], and vasculature [8], [12].

PDGF-dependent mitogenic pathways have been implicated in the pathogenesis of astrocytomas [13], as both PDGF A and B proteins are expressed in malignant astrocytomas. Less is known about the recently-identified PDGF-C and –D isoforms [14], [15], [16], but PDGF-C mRNA has been detected in several glioma cell lines [17], and it is evidently able to induce de-differentiation of astrocytes [18]. In addition, PDGF-C has been reported to induce angiogenic activity indirectly, via upregulation of VEGF [16], [19], and directly, via activation of PDGF-Rα and Rα−Rβ receptors [20]. These observations, along with the fact that PDGF-Rα and -Rβ are expressed in astrocytomas [21] and glioma endothelial cells [22] point to PDGF-C as a potentially important mediator of brain tumor progression. In this study, we over- and under-expressed PDGF-C in a human glioma line U87MG to assess the role of PDGF-C in glioma angiogenesis and in anti-VEGFR-2-induced vessel normalization.

## Materials and Methods

### Ethics Statement

All animal experiments performed in this study were conducted in compliance with the guidelines of the Office of Live Animal Research at Massachusetts General Hospital.

### PDGF-C over expression

To overexpress PDGF-C, the full-length cDNA was cloned into a peak12 vector driven by the EF1-a promoter (obtained from Dr. Brian Seed, Massachusetts General Hospital, Boston, MA). This expression vector was stably transfected into U87MG cells using LipofectAMINE 2000 (Invitrogen, Carlsbad, CA) according to the manufacturer's protocol. Transfected cells were selected with 0.5 µg/ml puromycin. Because of the lack of the necessary activating enzymes contained in normal fetal calf serum, plasmin digestion was used to demonstrate the proteolytic removal of the CUB domain confirming that the PDGF-C protein would be functional.

### RNA interference

PDGF-C down regulation was achieved using the pSilencer hygro (Ambion, Austin, TX) expression vector. The sequences for the PDGF-C shRNAi were taken from Genbank accession no. NM_016205. The coding sequences for shRNAi started with AA and were chosen so that there was no significant sequence homology with other genes, especially other PDGF family genes, assessed via Basic Local Alignment Search Tool (BLAST) analysis (http://blast.ncbi.nlm.nih.gov/blast). Hairpin shRNAi-encoding oligonucleotides were allowed to anneal and then ligate into the vector according to the manufacturer's protocol. Bacteria were transformed with the vector and screened using a restriction digest to ensure plasmid uptake. The plasmid was amplified and transfected into U87MG cells using LipofectAMINE 2000. Transfected cells were selected using hygromycin and clones were screened using western blot analysis to demonstrate lower protein levels due to RNA interference. “Scrambled” shRNA was used as the mock transfection control.

### Animal model and treatment

Cranial windows were implanted into 8–10 week old male SCID mice as previously described [23], [24]. After one week, a single cell suspension (200,000 cells) of U87MG, U87-C (PDGF-C transfected variant) or U87si (shRNAi-transfected variant) was implanted under the window into the cerebral cortex at a depth of approximately 0.4 mm. Treatment with the anti-VEGFR2 antibody DC101 (ImClone System Inc., New York, NY) was started as soon as the tumors reached a mean diameter of 2.0 to 2.5 mm, and this was designated day 0. DC101 was administered i.p. (40 mg/kg) every third day for a maximum of three doses (day 0, 3, and 6); a control group with size-matched tumors received non-specific rat IgG at 40 mg/kg on a similar schedule.

### Multiphoton Laser Scanning Microscopy (MPLSM) Angiography

In vivo MPLSM angiography of glioblastoma vessels was performed as described previously [24]. For each tumor, four adjacent images were obtained through the cranial window at day 0 (before treatment), and the same regions were revisited and recorded at day 2, 5, and 8. For each region, 41 images spaced 5 microns apart were collected in the ***z*** direction, producing ∼512×512×200 µm volume stacks. A maximum intensity projection of images 16 to 41 was created, representing the region from 75 µm to 200 µm under the brain surface. Image analysis was performed as described previously [25].

### Vessel wall analysis

Brains of tumor-bearing mice were harvested three weeks (±5 days) after implantation when tumors reached a mean diameter of 2.5 (±1) mm. Perfused vessels, were labeled by injection of biotinylated Lycopersicon Esculentum lectin and the tissue was fixed by perfusion of 4% paraformaldehyde [26]. Brains were embedded in OCT and stored at −80°C. Frozen sections (10 µm) were incubated with a streptavidin-conjugated Alexa 488 to visualize the lectin (Molecular Probes Inc., Eugene, OR) and stained with one of the following Chemicon primary antibodies: rabbit anti-Collagen IV (1∶2000; AB756), rabbit anti-NG2 (1∶1000; AB5320), or rabbit anti-Desmin (1∶200). For quantification, five well-vascularized regions per section were randomly selected and imaged using identical microscope settings. Pericyte coverage in control tumors and PDGF-C overexpressors was assessed by the intensity profile of pericyte staining near the vascular endothelium: the fraction of NG2- or desmin- positive pixels was analyzed at various distances from the vessel wall, and the average signal for each animal (based on 5 areas) is plotted.

### Cell Proliferation Assay

Cell proliferation was measured using Cell Titer 96® Aqueous One Solution obtained from Promega (Madison, Wisconsin). Cells were washed with PBS, trypsinized, and counted using the trypan blue exclusion assay. Cells were seeded at different densities in a 96 well plate and allowed to proliferate for 18 hours. The media was then changed and 20 µl of Cell Titer MTS solution was added to each well. After two additional hours, absorbance was read at 490 nm on a plate reader. Each seeding density was run in triplicate and the experiment was repeated three times.

### Cell migration Assay

Calcein AM (Molecular Probe, Eugene, OR) –labeled cells were seeded in laminin pre-coated Fluoroblock™ filter wells (Becton Dickinson, Palo Alto, CA). 6×10^4^ cells suspended in 300 µl media were placed into each well insert. Image analysis quantified the rate of cell migration through the 8 µm pores to the bottom of the filter, based on images acquired from the bottom of each well 1, 2, 3, 6, 12, 24 hours after seeding.

### Receptor and transcript analysis

Wild-type and stably-transfected U87 cells were cultured in serum-free medium overnight. Media were collected and protein concentration determined. In total, 35 mg protein was precipitated using ice-cold 10% trichloroacetic acid (TCA). Precipitated proteins were washed several times with 80% ethanol and then subjected to SDS–PAGE using 12% polyacrylamide gels (BioRad) under reducing conditions. Immunoblotting was performed using PDGF-C (1∶1,000, R&D Systems Minneapolis, MN), PDGFRa (1∶1,000, Cell Signaling, Beverly, MA) and phospho-PDGFRa (1∶1,000, Cell Signaling, Beverly, MA) antibodies. Bound antibodies were detected using Enhanced Chemiluminiscence Plus reagent (ECL+, Amersham).

### Plasmin Digestion

Conditioned Media samples were digested with 0.5–1 units per ml of plasmin in 40 mM Tris-HCl, 2 mM CaCl_2_, 2 mM MgCl_2_, and 0.02% Tween-20 for 30 minutes at 37°C. Western blot analysis using 15% polyacrilamide gels demonstrated removal of the PDGF-C CUB domain; this removal is essential for binding of PDGF-C to its receptors.

### Vessel Network Analysis

Vessel networks were quantified as described previously [25]. In summary, the tracing algorithm begins by extracting a set of candidate seeds over a sparse grid. Then, a super-ellipsoid model is fit at each seed. If a vessel is detected, tracing proceeds in the forward/reverse directions using the locally estimated pose. During tracing, the model is restricted to moving in the normal plane perpendicular to the medial axis of the best-fit super-ellipsoid. Tracing continues until the trace hits the boundary, another vessel, or the likelihood ratio falls below a predetermined threshold. The tracing results provide a vectorized backbone of the vascular bed including topological information such as branch points and the number of vessel segments.

### Microvascular Permeability Measurement

Mice were first injected with a bolus (0.1 ml) of 10 mg/ml fluorescein-labeled Dextran (2 M MW; Sigma) i.v., and angiograms where obtained in a well-perfused tumor area using the 20× objective lens of an epifluorescence microscope. Thereafter, 1% tetramethylrhodamine-labeled BSA (Molecular Probes, Eugene, OR) was injected, and rhodamine fluorescence intensity of the tumor tissue was measured every 2 min for a total of 20 min by a photomultiplier (9203B; EMI, Rockaway, NJ). The microvascular permeability to albumin was then calculated from the change in intensity as previously described [27].

### Statistical analysis

Data are expressed as mean±standard error of the mean. The principle statistical test was the Mann-Whitney U test; p<0.05 was considered to be statistically significant.

## Results

### Human gliomas express significant levels of PDGF-C

**Figure 1:**
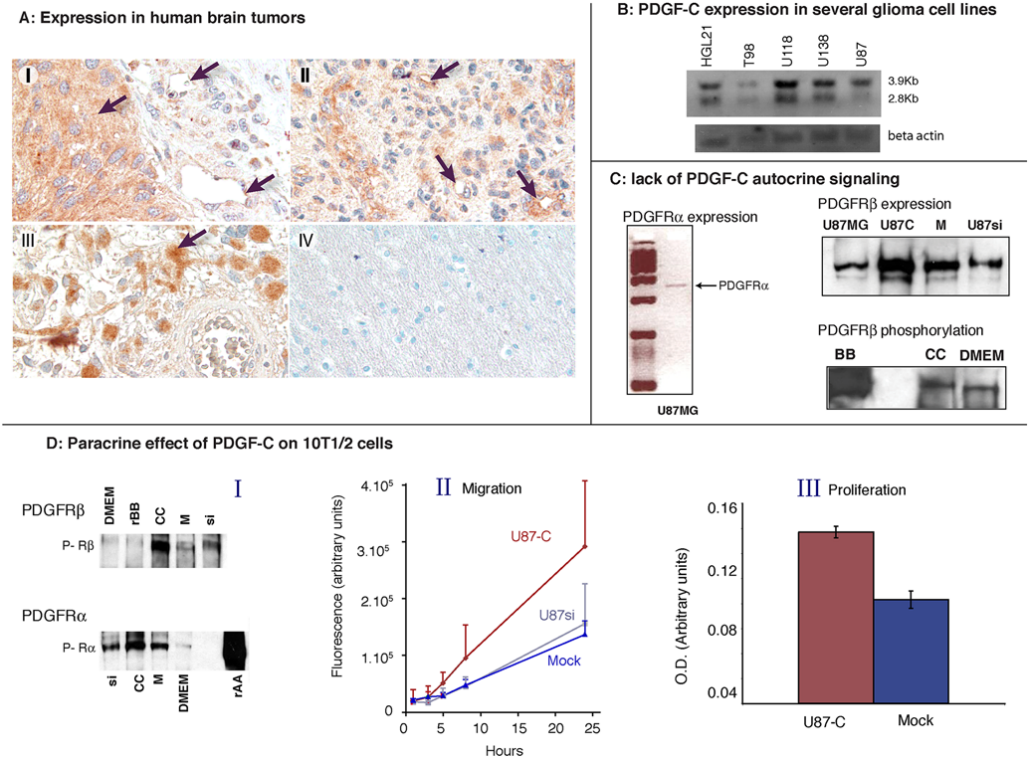
PDGF-C expression in human gliomas – autocrine & paracrine signaling. A: Expression of PDGF-C in human samples of gliomas. 27 samples of Grade IV glioma (Tissue Microarrays, kind gift from Dr. David Louis, MGH) were screened for expression of PDGF-C using immunohistochemistry (PDGF-C monoclonal antibody from R&D, used at 1/100). 23 out of 27 specimens expressed PDGF-C in tumor cells and/or in the peri-vascular area. Panels I, II and III are 3 different patients; panel IV is normal brain tissue present on the same array. In I and III positive tumor cells can clearly be identified; positive endothelium is evident in I and II, and perivascular cells also stain in panel II. In all panels, brown color (DAB) shows positive labeling for PDGF-C while blue (hematoxilin) stains nuclei. B: PDGF-C RNA expression in several common glioma cell lines. We chose U87 since it has a relatively low level expression compared to other cell lines and is thus amenable to downreglation. C: Lack of autocrine effect of PDGF-C on U87MG cells. Qt-RTPCR of PDGFRα in U87 cells shows that RNA is present, but there is no protein detectable by Western blot (not shown). PDGFRβ protein expression in parental (U87MG), PDGF-C overexpressor (U87-C), Mock-transfected (M) or ShRNAi cells (si). PDGFRβ phosphorylation. Cells were treated overnight with one of the following: recombinant PDGF-B (rBB, positive control); conditioned medium from cells overexpressing PDGF-C (CC); fresh DMEM culture medium (DMEM). PDGF-C did not induce phosphorylation above the baseline level. D: Effect of PDGF-C on 10T1/2 myofibroblasts. Panel I, Top: Phosphorylation of PDGFRβ. Cells were stimulated overnight with fresh DMEM culture medium (DMEM), recombinant PDGFBB (rBB; positive control), U87-C conditioned medium (CC), or conditioned medium from mock-transfected cells (M). PDGF-C did not stimulate this receptor. Bottom: phosphorylation of PDGFRα on 10T1/2 cells in culture. Cells were stimulated overnight with conditioned medium from U87si (si), U87-CC (CC), mock transfected cells (M), or fresh DMEM culture medium (DMEM). Recombinant PDGFAA was used as a positive control (rAA). The phosphorylation induced by the mock and U87si media is likely due to PDGF-A produced by these cells. Treatment of 10T1/2 cells with PDGF-C conditioned medium results in faster migration (Boyden chamber assay; panel II) and proliferation (WST-1 assay; panel III).

To assess whether PDGF-C is expressed in human glioblastomas, we first examined a series of 27 surgical specimens of human glioblastomas from MGH patients. In 23 of these samples, we detected PDGF-C in cancer cells but also in endothelial and perivascular cells (Figure 1A). In contrast, there was no detectable PDGF-C in normal brain; this suggested that PDGF-C might play a role in human gliomas.

#### PDGF-C affects myofibroblasts, but not U87 cells, in vitro

We then developed PDGF-C over- and under-expressing glioma cells. After screening five common glioma cell lines (T98G, HGL21, U87, U373, U138) for expression of PDGF-C (see Figure 1B), we chose U87MG as the parental line because of its relatively modest levels of PDGF-C, which made it possible to effectively down-regulate, as well as over-express PDGF-C in this line.

Since some gliomas express the PDGF-C receptors PDGFRα and PDGFRβ [4], [28] we checked for autocrine signaling in our U87MG cells. Although PDGFRα protein was undetectable in the parental or transfected U87MG cells by western blot analysis, RT-PCR confirmed a low level of expression (Figure 1C). The other receptor, PDGFRβ, was detected by Western blot, but its expression level was the same in the U87-C, U87-mock or U87si cell lines. Even in the U87-C cells, which produce high levels of PDGF-C, there was only a background level of PDGFRβ phosphorylation, indicating a lack of autocrine signaling (Figure 1C). There was also no significant difference in proliferation or migration between the parental and transfected cells in culture. Consequently, PDGF-C did not produce an autocrine effect on any of the U87 variants.

Angiogenesis array analysis of the three cell types revealed that overexpression or downregulation of PDGF-C had no effect on RNA levels of other angiogenic molecules such as VEGFA, Angiopoietins, IL8 and neuropilin 1. In addition, western blot analysis confirmed that levels of PDGF-A and PDGF-B, which are both expressed in U87 cells, were not changed by PDGF-C transfection.

PDGF signaling has been implicated in mural cell migration and differentiation [29]. To check whether our cells induce myofibroblast proliferation or migration, we applied conditioned medium (CM) to 10T1/2 cells, and found that proliferation and migration rates were significantly increased when the cells were stimulated with U87-C CM compared to Mock or U87si CM (Figure 1D).

### PDGF-C stabilizes tumor vessels

A hallmark of gliomas is abnormal vascularization. Typically, blood vessels in these tumors are large, tortuous, leaky, and they have fewer perivascular cells and abnormal basement membrane [24], [30]. We have shown that anti-angiogenic therapy can transiently normalize glioma vessels, decreasing vessel densities, diameters and permeability, and inducing a more normal basement membrane and wall structure [24], [30]. This normalization can actually prolong survival by decreasing the edema associated with the abnormal vasculature [30].

**Figure 2:**
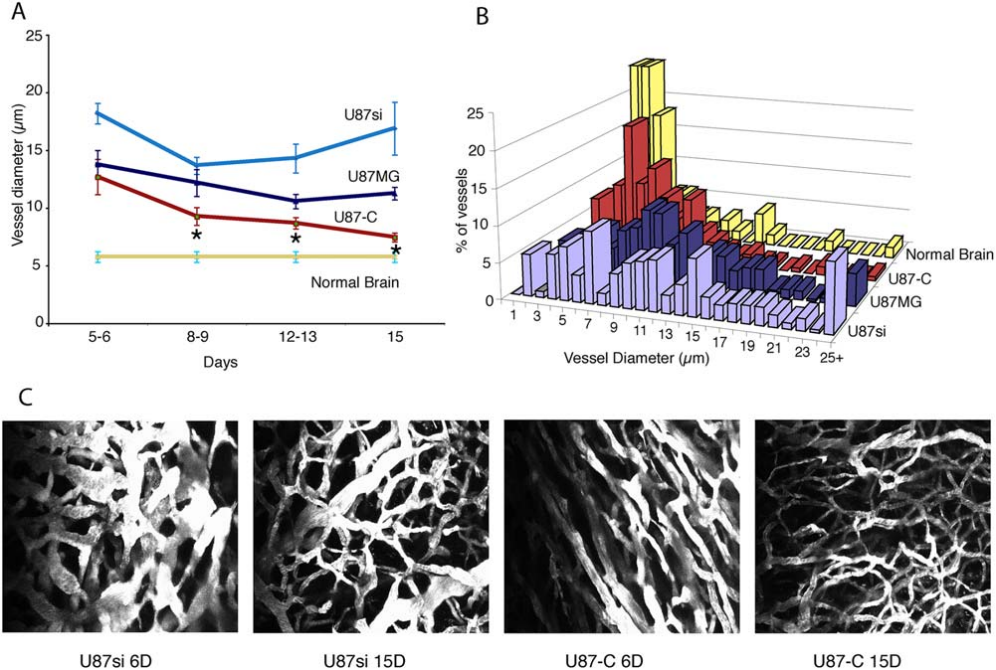
Blood vessel morphology in brain tumors. A: PDGF-C expression causes a decrease in vessel diameter over 15 days of growth. B: At day 15 after implantation, the vessel size distribution in the PDGF-C over-expressing tumors resembles that of normal brain, while the siRNA and parental tumors have distributions shifted to higher diameters, with significant numbers of large-diameter (>25 µm) vessels. C: representative 2-photon images of the vessel networks.

To check whether PDGF-C is involved in vessel abnormalities or normalization, we grew tumors from single cell suspensions in the brain parenchyma of immunodeficient mice bearing transparent cranial windows and performed longitudinal intra-vital analysis [23]. Fluorescent angiography revealed dramatic differences in tumor vasculature that varied with the level of PDGF-C produced by the tumors. By day 15 after implantation, vessels in PDGF-C overexpressing tumors were more normal, with relatively small diameters (Figure 2). In contrast, U87si had larger blood vessels (Figure 2A and 2C). In addition, the distributions of vessel sizes were quite different; for example, the U87si and parental tumors had more large-diameter vessels (>25 µm) than the U87-C tumors (Figure 2B).

**Figure 3:**
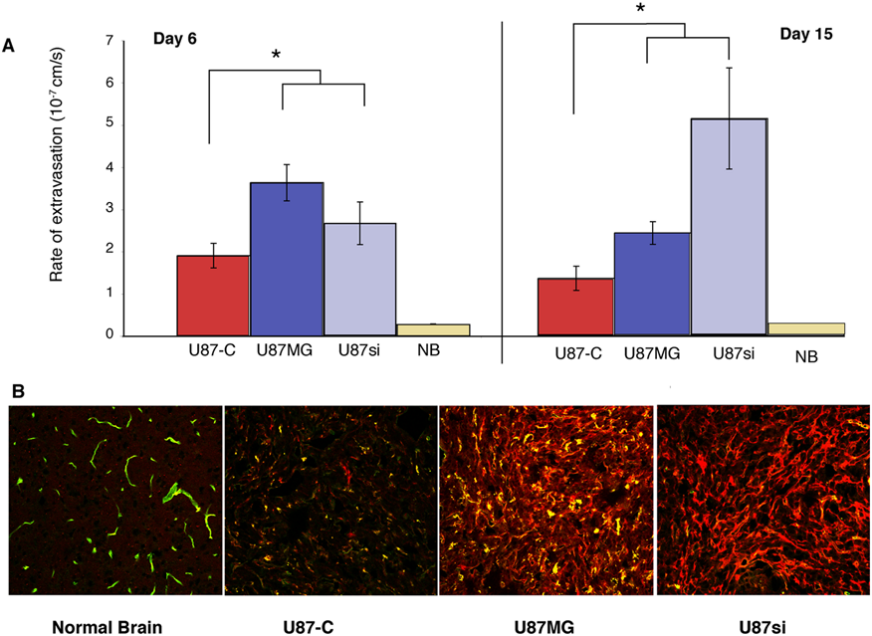
PDGF-C affects the transvascular flux of BSA. A: Effective vascular permeability was measured 6 days and 15 days after tumor implantation. The vessels in PDGF-C overexpressing tumors have lower permeability both 6 and 15 days following implantation. Normal brain (NB) vessels are relatively impermeable. B: Delivery of the BSA marker (red) is inhibited by PDGF-C overexpression (Green = CD31).

PDGF-C also affected vessel *function*. Permeability analysis [24] showed that tumor vessels in U87-C tumors were less permeable than the parental or U87RNAi tumors at both 6 and 15 days after implantation (Figure 3A). The decreased vessel leakiness likely contributed to the poor distribution of the fluorescent BSA throughout the U87-C tumors compared to the parental and U87RNAi- tumors (Figure 3B). It is also possible, however, that PDFG-C affected interstitial transport, hindering penetration. The differences in permeability could not be attributed to differences in VEGFA levels, as there were no significant differences in VEGF expression by RT-PCR (either tumor- or host- produced) between U87-C, U87si and mock-transfected tumors.

**Figure 4:**
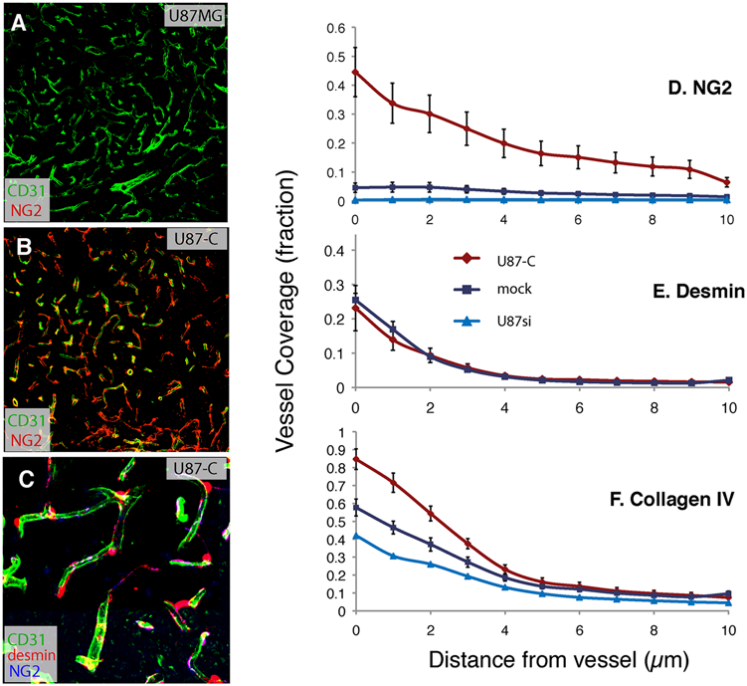
Changes in wall structure in response to PDGF-C overexpression. NG2 expression detected by confocal imaging of U87MG (A) and U87-C (B) tumors using immunohistochemistry on frozen sections of tumors harvested after 3 weeks post-implantation. Red pseudocolor represents NG2 positive cells, green pseudocolor represents CD31 positive endothelial cells. Panel C shows staining for NG2 (blue), desmin (red) and CD31 (green) on a 100 µm projection acquired with a confocal microscope. Panels D and E: quantification of NG2 and desmin, respectively. PDGF-C expression does not affect desmin-positive cell recruitment, but dramatically increases the number of NG2-positive cells around vessels. F: collagen IV distribution around vessels. The basement membrane is typically thicker than normal in U87MG [24] but U87-C displays an even thicker basement membrane.

Because vessel structure and function can be affected by association with perivascular cells, we next analyzed coverage of the tumor vessels by NG2- and desmin-positive pericytes. More of the U87-C tumor vessels were covered by NG2-positive perivascular cells (Figure 4A, B), and these cells extended more than 10 µm away from the vessels (Figure 4D). On the other hand, PDGF-C did not affect the distribution of desmin-positive perivascular cells: the parental and U87-C tumors had similar coverage by these cells, and in both cases, the cells were closely associated with the endothelium (distance less than ∼4 µm; Figure 4E). This indicates that PDGF-C recruited more perivascular cells to the vessel wall, and the expression patterns show that either the recruited cells expressed NG2 predominately, or else PDGF-C induced expression of NG2 in desmin-positive cells already close to the wall.

The dramatic changes in pericyte investment induced by PDGF-C were accompanied by differences in the vascular basement membrane. U87-C tumor vessels had greater coverage of collagen IV, and the collagen IV layer was thicker, extending farther from the vessel wall (Figure 4F). These changes in wall structure likely contributed to the differences in tumor vessel function.

### Overexpression of PDGF-C confers resistance to anti-VEGF therapy

**Figure 5:**
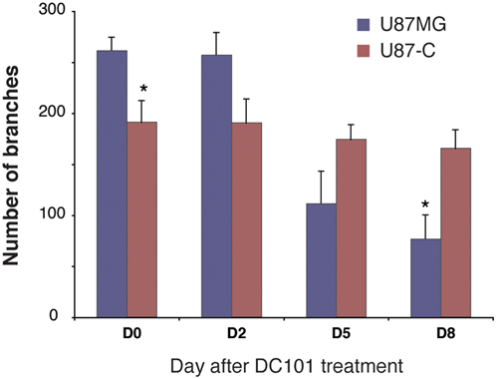
PDGF-C attenuates the response to VEGFR2 blockade. U87MG or U87-C tumor-bearing mice were treated with DC101 antibody (40 mg/kg) when their tumors reached 2 mm in diameter. Intravenous injections of DC101 were performed on days 0, 3 and 6. Vessels in the parental U87MG tumors are pruned in response to DC101 (fewer branch points). In contrast, U87-C tumors showed no response (n = 6).

It has been suggested that increased pericyte fortification can render vessels insensitive to anti-VEGF therapy [31]. To assess whether the changes in wall structure induced by PDGF-C affect the response to anti-VEGF therapy, we treated tumor-bearing animals with the anti-VEGFR-2 antibody DC101. In our prior study, this dose and schedule of DC101 induced vessel normalization and modest tumor regression in U87 tumors [24]. On day zero (just before the initial treatment), the PDGF-C tumors had lower vessel density compared to the parental tumors (Figure 5); with treatment, however, the parental tumor vessel density decreased, while the number of branches in the U87-C tumor remained virtually unchanged. This suggests that PDGF-C protects tumor vessels from DC101-induced pruning.

### Treatment with DC101 increases PDGF-C expression in host cells

**Figure 6:**
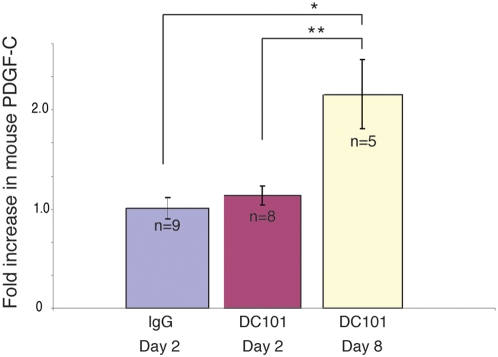
DC101 increases host PDGF-C expression. U87MG tumor-bearing mice were treated with DC101 (40 mg/kg) when their tumors reached 2 mm in diameter. Tumors were harvested and snap-frozen at baseline (n = 9), 2 days (n = 8) and 8 days (n = 5) after initiation of treatment. Day 8 corresponds to the time of escape from DC101 treatment in this model[24]. Quantitative PCR was performed using primers for mouse PDGF-C (forward primer: ACCACGAGTCCTTCGGTGTT, located at 529 bp; reverse primer: GCATTGTTGAGCAGGTCCAA, located at 566 bp). At day 8 DC101-treated tumors had significantly more mouse PDGF-C compared to base line and to control, baseline tumors (p<0.05) IgG-treated animals die before day 8.

It has been shown that in murine tumors resistant to anti-VEGF treatment, tumor associated fibroblasts (TAFs) increase their production of PDGF-C to sustain angiogenesis [32]. To assess whether this pathway operates in our system we quantified mRNA levels for murine PDGF-C in tumor-bearing animals (wild type U87MG) treated with DC101. Eight days after initiation of the treatment murine PDGF-C in the tumor extract was significantly increased (p = 0.041) compared to control brain (Figure 6) confirming that PDGF-C is possibly involved in resistance to anti-VEGFR2 therapy in brain tumors as well. In contrast murine VEGF-A, human VEGF-A and PDGF-C did not increase.

## Discussion

PDGF-C is evidently involved in many aspects of stromal dynamics. It has been shown to mediate renal fibrosis [33], to be secreted by Wilm's tumor cells [34], to be a potential oncogene in malignant pleural mesothelioma [35] and to recruit stromal cells during carcinogenesis in lung [36] and liver [37]. Its role in brain tumors has been less studied, but its potential importance was highlighted by studies from Lokker and coworkers, showing a potential autocrine role of PDGF-C in gliomas [17].

In the current study we established that PDGF-C plays a role in glioma progression through anti-VEGF therapy. We found that over expression of this growth factor changes the morphology and function of the brain vessels, rendering them more stable and less sensitive to anti-VEGFR-2 inhibition.

We specifically chose U87 as the parental cell line because of its lack of PDGF-C autocrine signaling (possibly because these cells express very little PDGFRα); this allowed us to focus on the paracrine effects on the vasculature. However, a recent survey of patient archival biopsies showed that 93% of GBMs had detectable levels of PDGFRα and 37% had detectable PDGFRβ on tumor cells, while 93% and 100% had detectable levels in blood vessels, respectively [4]. Therefore, future studies should assess the presence and implications of autocrine signaling in gliomas, and the potential benefits of inhibitors that also target the PDGF receptor, such as Cediranib. We expect that the vascular effects demonstrated here will still exist in tumors with autocrine PDGF-C signaling, but they might be accompanied by more aggressive cancer cell proliferation and migration.

Studies of the involvement of PDGF-C in ischemic stroke have shown that it increases the permeability of the blood brain barrier by acting on perivascular astrocytes in response to tissue plasminogen activator [38]. This result conflicts with our decrease in permeability with PDGF-C expression, and may be due to the chronic expression of PDGF-C in our tumor model, which contrasts with the abrupt perturbations caused by strokes in otherwise healthy brain tissue. In this case tPA activation of the latent PDGF-C activates PDGFRα/β and increases permeability. This could be blocked by administration of a PDGFR blocker, such as, imatinib. In tumor tissue, however, inhibition of PDGFR has been shown to induce the opposite effect, increasing permeability, and increasing fluid retention and ascites in cancer patients [39], [40]. These contrary responses are likely due to differences in the structure of tumor vessels compared to normal brain vessels.

We propose that, in our system, expression of PDGF-C drives the recruitment and differentiation of perivascular cells, resulting in better basement membrane integrity and a more mature, stable vascular wall with a lower permeability. Indeed, it has been shown that brain and peripheral nerve pericytes can contribute directly to vessel wall barrier function via pericyte-pericyte junctions [41]. Therefore, high levels of PDGFC might render tumors more difficult to treat with anti-VEGF therapy by stabilizing vessels, decreasing transvascular transport and minimizing their dependence on VEGF for survival. In addition, there appears to be communication between these pathways, as treatment with VEGFR2 antibody increased PDGF-C production by the host cells.

The role of PDGF-C appears to vary significantly depending on the microenvironment. A recent study of tumor associated fibroblast (TAF) involvement in subcutaneous tumor angiogenesis concluded that TAFs produce PDGF-C to direct the process of endothelial cell migration and angiogenesis, independent of VEGF [32]. On the other hand, another group recently showed that PDGF-C secreted by melanoma tumor cells helps recruit cancer associated fibroblasts, which in turn, produce a tumor-promoting growth factor [42]. In our study we found that PDGF-C produced by tumor cells can recruit pericytes, stabilizing vessels and inhibiting DC101-induced pruning. These seemingly divergent results are likely due to the lack of fibroblasts in brain tumors. It is also possible that the discrepancy is related to differences in local chemotactic gradients, which might be overwhelmed by global expression of PDGF-C by the cancer cells. These considerations underscore the need for a more fundamental understanding of how this growth factor contributes to tumor physiology in various growth sites.

Brain tumors have recently been the focus of trials with various inhibitors of VEGF [43], which have had limited success– most patients progress through these therapies. Future studies should investigate whether PDGF-C is involved in the resistance to anti-VEGF therapy in the clinic, but this will only become possible as patients enter clinical trials with up-front anti-VEGF treatment with the possibility of resection at the time of recurrence, thereby providing surgical specimens to address this important question.

## References

[1] Jain RK, di Tomaso E, Duda DG, Loeffler JS, Sorensen AG (2007). Angiogenesis in brain tumours.. Nat Rev Neurosci.

[2] Cloughesy TF, Prados MD, Wen PY, Mikkelsen T, Abrey LE (2008). A phase II, randomized, non-comparative clinical trial of the effect of bevacizumab alone or in combination with irinotecan (CPT-11) on 6-month progression free survival in recurrent, treatment-refractory glioblastoma.. J Clin Oncol.

[3] Vredenburgh JJ, Desjardins A, Herndon JE, Marcello J, Reardon DA (2007). Bevacizumab plus irinotecan in recurrent glioblastoma multiforme.. J Clin Oncol.

[4] Batchelor TT, Sorensen AG, di Tomaso E, Zhang WT, Duda DG (2007). AZD2171, a pan-VEGF receptor tyrosine kinase inhibitor, normalizes tumor vasculature and alleviates edema in glioblastoma patients.. Cancer Cell.

[5] George D (2003). Targeting PDGF receptors in cancer–rationales and proof of concept clinical trials.. Adv Exp Med Biol.

[6] Kaplan DR, Chao FC, Stiles CD, Antoniades HN, Scher CD (1979). Platelet alpha granules contain a growth factor for fibroblasts.. Blood.

[7] Funa K, Papanicolaou V, Juhlin C, Rastad J, Akerstrom G (1990). Expression of platelet-derived growth factor beta-receptors on stromal tissue cells in human carcinoid tumors.. Cancer Res.

[8] Heldin CH, Westermark B (1999). Mechanism of action and in vivo role of platelet-derived growth factor.. Physiol Rev.

[9] Bhardwaj B, Klassen J, Cossette N, Sterns E, Tuck A (1996). Localization of platelet-derived growth factor beta receptor expression in the periepithelial stroma of human breast carcinoma.. Clin Cancer Res.

[10] Kawai T, Hiroi S, Torikata C (1997). Expression in lung carcinomas of platelet-derived growth factor and its receptors.. Lab Invest.

[11] Sundberg C, Branting M, Gerdin B, Rubin K (1997). Tumor cell and connective tissue cell interactions in human colorectal adenocarcinoma. Transfer of platelet-derived growth factor-AB/BB to stromal cells.. Am J Pathol.

[12] Jain RK, Lahdenranta J, Fukumura D (2008). Targeting PDGF signaling in carcinoma-associated fibroblasts controls cervical cancer in mouse model.. PLoS Med.

[13] Li J, Perry A, James CD, Gutmann DH (2001). Cancer-related gene expression profiles in NF1-associated pilocytic astrocytomas.. Neurology.

[14] Bergsten E, Uutela M, Li X, Pietras K, Ostman A (2001). PDGF-D is a specific, protease-activated ligand for the PDGF beta-receptor.. Nat Cell Biol.

[15] LaRochelle WJ, Jeffers M, McDonald WF, Chillakuru RA, Giese NA (2001). PDGF-D, a new protease-activated growth factor.. Nat Cell Biol.

[16] Li X, Ponten A, Aase K, Karlsson L, Abramsson A (2000). PDGF-C is a new protease-activated ligand for the PDGF alpha-receptor.. Nat Cell Biol.

[17] Lokker NA, Sullivan CM, Hollenbach SJ, Israel MA, Giese NA (2002). Platelet-derived growth factor (PDGF) autocrine signaling regulates survival and mitogenic pathways in glioblastoma cells: evidence that the novel PDGF-C and PDGF-D ligands may play a role in the development of brain tumors.. Cancer Res.

[18] Dai C, Celestino JC, Okada Y, Louis DN, Fuller GN (2001). PDGF autocrine stimulation dedifferentiates cultured astrocytes and induces oligodendrogliomas and oligoastrocytomas from neural progenitors and astrocytes in vivo.. Gene and Development.

[19] Li X, Eriksson U (2003). Novel PDGF family members: PDGF-C and PDGF-D.. Cytokine Growth Factor Rev.

[20] Cao R, Brakenhielm E, Li X, Pietras K, Widenfalk J (2002). Angiogenesis stimulated by PDGF-CC, a novel member in the PDGF family, involves activation of PDGFR-alphaalpha and -alphabeta receptors.. Faseb J.

[21] Kilic T, Alberta JA, Zdunek PR, Acar M, Iannarelli P (2000). Intracranial inhibition of platelet-derived growth factor-mediated glioblastoma cell growth by an orally active kinase inhibitor of the 2-phenylaminopyrimidine class.. Cancer Res.

[22] Hermansson M, Nister M, Betsholtz C, Heldin CH, Westermark B (1988). Endothelial cell hyperplasia in human glioblastoma: coexpression of mRNA for platelet-derived growth factor (PDGF) B chain and PDGF receptor suggests autocrine growth stimulation.. Proc Natl Acad Sci U S A.

[23] Jain RK, Munn LL, Fukumura D (2002). Dissecting tumour pathophysiology using intravital microscopy.. Nature Rev Cancer.

[24] Winkler F, Kozin SV, Tong RT, Chae SS, Booth MF (2004). Kinetics of vascular normalization by VEGFR2 blockade governs brain tumor response to radiation: role of oxygenation, angiopoietin-1, and matrix metalloproteinases.. Cancer Cell.

[25] Tyrrell JA, di Tomaso E, Fuja D, Tong R, Kozak K (2007). Robust 3-D modeling of vasculature imagery using superellipsoids.. IEEE Trans Med Imaging.

[26] di Tomaso E, Capen D, Haskell A, Hart J, Logie J (2005). Mosaic tumor vessels: cellular basis and ultrastructure of focal regions lacking endothelial cell markers.. Cancer Research.

[27] Yuan F, Salehi HA, Boucher Y, Vasthare US, Tuma RF (1994). Vascular permeability and microcirculation of gliomas and mammary carcinomas transplanted in rat and mouse cranial windows.. Cancer Res.

[28] Black P, Carroll R, Glowacka D (1996). Expression of platelet-derived growth factor transcripts in medulloblastomas and ependymomas.. Pediatr Neurosurg.

[29] Gerhardt H, Betsholtz C (2003). Endothelial-pericyte interactions in angiogenesis.. Cell Tissue Res.

[30] Kamoun W, Ley C, Farrar C, Duyvernman A, Lahdenranta J (in press). Edema control by cediranib, a VEGF targeted kinase inhibitor, prolongs survival despite persistent brain tumor growth in mice.. JCO.

[31] Bergers G, Hanahan D (2008). Modes of resistance to anti-angiogenic therapy.. Nat Rev Cancer.

[32] Crawford Y, Kasman I, Yu L, Zhong C, Wu X (2009). PDGF-C mediates the angiogenic and tumorigenic properties of fibroblasts associated with tumors refractory to anti-VEGF treatment.. Cancer Cell.

[33] Eitner F, Bucher E, van Roeyen C, Kunter U, Rong S (2008). PDGF-C is a proinflammatory cytokine that mediates renal interstitial fibrosis.. J Am Soc Nephrol.

[34] Fraizer GE, Bowen-Pope DF, Vogel AM (1987). Production of platelet-derived growth factor by cultured Wilms' tumor cells and fetal kidney cells.. J Cell Physiol.

[35] Gordon GJ, Rockwell GN, Jensen RV, Rheinwald JG, Glickman JN (2005). Identification of novel candidate oncogenes and tumor suppressors in malignant pleural mesothelioma using large-scale transcriptional profiling.. Am J Pathol.

[36] Tejada ML, Yu L, Dong J, Jung K, Meng G (2006). Tumor-driven paracrine platelet-derived growth factor receptor alpha signaling is a key determinant of stromal cell recruitment in a model of human lung carcinoma.. Clin Cancer Res.

[37] Campbell JS, Johnson MM, Bauer RL, Hudkins KL, Gilbertson DG (2007). Targeting stromal cells for the treatment of platelet-derived growth factor C-induced hepatocellular carcinogenesis.. Differentiation.

[38] Su EJ, Fredriksson L, Geyer M, Folestad E, Cale J (2008). Activation of PDGF-CC by tissue plasminogen activator impairs blood-brain barrier integrity during ischemic stroke.. Nat Med.

[39] Jayson GC, Parker GJ, Mullamitha S, Valle JW, Saunders M (2005). Blockade of platelet-derived growth factor receptor-beta by CDP860, a humanized, PEGylated di-Fab', leads to fluid accumulation and is associated with increased tumor vascularized volume.. J Clin Oncol.

[40] Hagendoorn J, Tong R, Fukumura D, Lin Q, Lobo J (2006). Onset of abnormal blood and lymphatic vessel function and interstitial hypertension in early stages of carcinogenesis.. Cancer Res.

[41] Shimizu F, Sano Y, Maeda T, Abe MA, Nakayama H (2008). Peripheral nerve pericytes originating from the blood-nerve barrier expresses tight junctional molecules and transporters as barrier-forming cells.. J Cell Physiol.

[42] Anderberg C, Li H, Fredriksson L, Andrae J, Betsholtz C (2009). Paracrine signaling by platelet-derived growth factor-CC promotes tumor growth by recruitment of cancer-associated fibroblasts.. Cancer Res.

[43] Gerstner ER, Duda DG, di Tomaso E, Sorensen G, Jain RK (2007). Antiangiogenic agents for the treatment of glioblastoma.. Expert Opin Investig Drugs.

